# Supplementary material for: Complex interactions in healthcare expenditure through the years: A panel data analysis using fsQCA in OECD countries with policy implications
Source: PLoS One. 2025 May 21;20(5):e0324497. doi: 10.1371/journal.pone.0324497 (PMC12094791; doi:10.1371/journal.pone.0324497)
Supplement: Attachment — Submitted filename: Response to reviewers.pdf [file pone.0324497.s001.pdf]

Dear editor,

Thank you for your time and effort in reviewing my manuscript. I have carefully addressed each point raised by the reviewers and have made the necessary revisions accordingly. Below is a detailed response to each comment.

Response to Reviewer #1:

Comment: In Sub-section 2.2, the authors mentioned that "Missing data points were estimated using linear interpolation." It is recommended that this should be presented in more detail (including mathematical derivations) in the Appendix.

Response: To clarify this process, I have revised the manuscript as follows:

- I have explicitly stated that only a small portion of data is missing (<10%) by using the term "Missing values in isolated years."
- I have specified that the linear interpolation was implemented using the approx() function in R with rule=1 to ensure no extrapolation beyond the available data range.
- Additionally, I have cited the works of Moritz and Ragin to support the robustness of this method in handling missing data.

Response to Reviewer #2:

Comment 1: The authors should justify the placement of the sufficiency analysis process in the Results section, as it may be more appropriately included in the Methods section. Specifically, they should explain why the process of sufficiency analysis is presented in the Results section rather than in the Methods section. Additionally, clarification is needed on why certain study findings have been included in the Discussion section instead of the Results section.

Response: To improve the clarity and logical flow of the manuscript, I have made the following modifications:

1. The fsQCA analysis process, including calibration, necessity analysis, and sufficiency analysis, has been incorporated into the Methods section. This adjustment ensures that the presentation of sufficiency analysis in the Results section follows standard practice in fsQCA studies.
2. The overall structure of the manuscript has been revised to clearly distinguish between results and discussion. Previously, the structure was:

   2 Methods

   2.1 Model presentation and factors selection

   2.2 Method and data

   2.3 Calibration

   3 Results

   3.1 Necessity analysis results

   3.2 Sufficiency analysis results

   3.3 Panel data breakdown of fsQCA results

3.3.1 High-HCE
3.3.2 Low-HCE
4 Discussion

To enhance clarity, I have restructured it as follows:

2 Methods
2.1 Model presentation and factors selection
2.2 Method, data, and calibration
2.2.1 Method
2.2.2 Data
2.2.3 Calibration
3 fsQCA Analysis
3.1 Necessity analysis results
3.2 Sufficiency analysis results
4 Panel Data Breakdown of fsQCA Results
4.1 High-HCE
4.2 Low-HCE
5 Discussion

After the restructure, the Results section has been renamed to "Panel Data Breakdown of fsQCA Results," emphasizing that this section strictly presents the empirical findings of the panel data analysis.

3. To further clarify the distinction between fsQCA results and Discussion, I have added the following clarification sentence at the beginning of the Discussion section: *"Building on the fsQCA findings, this section analyzes causal configurations (recipes) and examines between-country (BECONS) and within-country (WICONS) consistencies through a comparative country-level lens."* This statement explicitly indicates that the Discussion section delves into a deeper interpretation of the fsQCA results rather than merely presenting them.

Comment 2: The manuscript should include line numbers to facilitate the review process.

Response: I have added line numbers to the manuscript to improve readability and ease the review process.

I appreciate the reviewers' valuable feedback, which has helped improve the clarity and structure of my manuscript. Thank you for your consideration, and I look forward to your further feedback.

Best regards,

Shanren Nie
Dong Liu
Sheng Chen
